# Nasal delivery of H5N1 avian influenza vaccine formulated with GenJet™ or in vivo-jetPEI^®^ induces enhanced serological, cellular and protective immune responses

**DOI:** 10.1080/10717544.2018.1450909

**Published:** 2018-03-15

**Authors:** Weiping Cao, Margarita Mishina, Samuel Amoah, Wadzanai P. Mboko, Caitlin Bohannon, James McCoy, Suresh K. Mittal, Shivaprakash Gangappa, Suryaprakash Sambhara

**Affiliations:** a Immunology and Pathogenesis Branch, National Center for Immunization and Respiratory Diseases, Atlanta, GA, USA;; b Influenza Division, Centers for Disease Control and Prevention, Atlanta, GA, USA;; c Battelle Memorial Institute, Atlanta, GA, USA;; d Department of Comparative Pathobiology, Purdue University, West Lafayette, IN, USA;; e Oak Ridge Institute for Science and Education (ORISE), CDC Fellowship Program, Oak Ridge, TN, USA;; f Department of Pathology and Laboratory Medicine, Emory University, Atlanta, GA, USA

**Keywords:** Cationic polymers, H5N1 vaccine, adaptive immunity, protective immunity, mouse model, influenza

## Abstract

Avian influenza virus infection is a serious public health threat and preventive vaccination is the most cost-effective public health intervention strategy. Unfortunately, currently available unadjuvanted avian influenza vaccines are poorly immunogenic and alternative vaccine formulations and delivery strategies are in urgent need to reduce the high risk of avian influenza pandemics. Cationic polymers have been widely used as vectors for gene delivery *in vitro* and *in vivo*. In this study, we formulated H5N1 influenza vaccines with GenJet™ or *in vivo*-jetPEI^®^, and showed that these formulations significantly enhanced the immunogenicity of H5N1 vaccines and conferred protective immunity in a mouse model. Detailed analyses of adaptive immune responses revealed that both formulations induced mixed T_H_1/T_H_2 antigen-specific CD4 T-cell responses, antigen-specific cytotoxic CD8 T-cell and memory B-cell responses. Our findings suggest that cationic polymers merit future development as potential adjuvants for mucosal delivery of poorly immunogenic vaccines.

## Introduction

Avian influenza viruses (AIVs) circulate naturally in wild aquatic birds and cause no illness in most cases. However, AIVs can infect domestic poultry, especially high pathogenic AIVs, and cause severe diseases with high mortality. Very rarely, human AIV infection occur through contact with infected animals or contaminated environments. Human infections with H5N1 viruses were first reported in Hong Kong in 1997 [Centers for Disease Control and Prevention (CDC), 1997]. As of April 2017, the H5N1 virus has spread to 18 countries resulting in 858 human infections with 53% mortality (World Health Organization, 2017). In recent years, H7N2, H7N9, H10N8, H6N1 and H5N6 viruses have crossed the species barrier to cause human infections (Gao et al., 2013; Shi et al., 2013; Zhang et al., 2014; Yang et al., 2015). Most AIVs caused sporadic and minor infections. However, novel mutations could potentially break the avian–human barrier and generate a serious pandemic; therefore, development of effective vaccines and exploration of new antiviral drugs are of urgent need. In 2007, a non-adjuvanted subvirion H5N1 avian influenza vaccine was approved by US Food and Drug Administration (FDA), but it requires two doses of 90 μg of hemagglutinin (HA), as compared to 15 μg of HA used for seasonal influenza vaccine. The two-dose regimen produced levels of antibodies expected to reduce the risk of H5N1 infection in 45% of people who received it (FDA, 2007). In 2013, an oil-in-water emulsion (AS03)-adjuvanted subviron H5N1 vaccine was approved in the US but only for stock-piling purpose (FDA, 2013). However, the continuing challenges including viral antigenic drift and shift, the high-dose vaccine requirement, the limited vaccine manufacturing capacity, and non-availability of proprietary adjuvants warrant developing novel formulations, adjuvants, and alternative delivery systems to improve the immunogenicity and protective efficacy of avian influenza vaccines.

Cationic polymers have been widely used as non-viral gene delivery vectors (Pack et al., 2005). The positive charge bearing cationic polymers interact with negative charged genetic material and form polyplexes spontaneously which protect the genes and mediate cellular entry (Pack et al., 2005). Cationic polymers have shown efficacy in DNA delivery as well as in sustained delivery of protein subunit vaccines (Adams et al., 2015). The cationic polymer polyethyleneimine (PEI) has been demonstrated as a potent mucosal adjuvant for viral glycoprotein antigens through promoting antigen uptake by dendritic cells (DCs) and subsequent DCs migration to draining lymph nodes (Wegmann et al., 2012). A recent study showed that avian H9N2 influenza whole inactivated virus plus PEI strongly enhanced mucosal and systemic antibody responses (Qin et al., 2015). GenJet™ and *in vivo*-jetPEI^®^ are two commercially available and widely used transfection agents. In our study, we used the poorly immunogenic A/IN/05 vaccine as an antigen and demonstrated that both GenJet™ and *in vivo*-jetPEI^®^ formulated H5N1 vaccine significantly enhance the protective immunity through the induction of mixed antigen-specific CD4 T_H_1/T_H_2 cells, antigen-specific cytotoxic CD8 cells, enhanced systemic antibody responses and memory B-cell responses.

## Material and methods

### Material

GenJet™ Plus DNA *In Vivo* Tranfection Reagent was purchased from SignaGen (Rockville, MD, USA) and *in vivo*-jetPEI^®^ was purchased from Polyplus Transfection (New York, NY). H5N1 Monovalent Influenza Split Vaccine (A/Indonesia/05/2005, A/IN/05) was provided by GlaxoSmithKline Vaccines (Ste-Foy, Quebec, Canada). RG H5N1 A/IN/05 virus was made by reverse genetics with HA and NA from A/IN/05 virus and the remaining six gene segments from A/Puerto Rico/8/1934 (PR8) virus. Viruses were propagated in 10-day-old embryonated chicken eggs for 48 h. Pooled allantoic fluids were clarified by centrifugation, aliquoted, and stored at –80 °C until use.

### Immunization and virus challenge

Six- to eight-week-old female BALB/c mice (Jackson Laboratories, Bar Harbor, ME) were immunized (five animals/group) by the intranasal (i.n.) route with 3 µg of A/IN/05 vaccine with or without GenJet™ (10 µl/each mouse as described earlier (Kulkarni et al., 2014)) or *in vivo*-jetPEI^®^ (0.6 μl/mouse according to manufacturer’s protocol). Control mice were immunized with GenJet™ alone, *in vivo*-jetPEI^®^ alone, or with PBS. Four weeks later, mice were boosted with the same immunization regimen. Sera were obtained three weeks post-primary and post-boost to determine antibody responses. Four weeks post-boost, mice were challenged with 5 × LD_50_ of RG A/IN/05 virus and monitored for weight loss and survival for 16 days. Animal research was conducted under the guidance of the CDC’s Institutional Animal Care and Use Committee in an Association for Assessment and Accreditation of Laboratory Animal Care International-accredited animal facility. Mice that lost >25% of their pre-infection body weight were euthanized.

#### ELISA

Nunc 96-well plates (Maxi-Sorb) were coated with 5 µg/ml HA of monovalent A/IN/05 vaccine in PBS at 4 °C overnight. The plates were then washed and blocked with 4% BSA in PBS-Tween (PBS-T) (0.05%) at room temperature (RT) for 1 h. Dilutions of sera were added onto the plate at RT for 2 h. After washing the plates, the HRP anti-mouse antibodies specific for IgA, IgG, IgM, IgG1, IG2a, IgG2b, or IgG3 (Southern Biotech, Birmingham, AL) were added onto the plate at RT for 1 h. The wells were developed with TMB peroxidase substrate (eBioscience, San diego, CA, USA) after washing and the absorbance was measured at 450 nm using a BioTek Plate Reader (BioTek, Winooski, VT, USA).

### ELISPOT assay

The frequency of virus-specific antibody-secreting cells (ASCs) in the spleen was detected by an ELISPOT assay. Briefly, 10^6^ cells were added onto antigen-coated plates and incubated overnight at 37 °C in a humidified atmosphere with 5% CO_2_. The plates were incubated with biotinylated anti-mouse IgG (Southern Biotech) followed by alkaline phosphatase-conjugated streptavidin and developed with Vector Blue Alkaline Phosphatase Substrate Kit III (Vector Laboratories, Burlingame, CA). Spot forming units were counted using ImmunoSpot^®^ (Cellular Technology Ltd., Shaker Heights, OH) and expressed as a percentage of antigen-specific IgG secreting B cells out of the total IgG-secreting B cells.

### Cell-mediated immune responses

Single cell suspensions were prepared from the lungs, draining lymph nodes and spleen tissues collected 1 week after the booster immunization. Activated T cells in the lung were identified using anti-CD44 antibody. HA-specific CD8^+^ T cells in the lungs, lymph nodes and spleens were identified using H-2K^d^/IYSTVASSL tetramer (NIH Tetramer Core Facility, Emory University, Atlanta, GA, USA). To detect intracellular cytokine production by CD4 or CD8 T cells, 10^6^ cells from the spleen were stimulated *in vitro* with RG A/IN/05 virus at an MOI of 1 for 16 h or with HA peptide (1 µM, NeoBiolab, Woburn, MA, USA) for 6 h, respectively. GolgiPlug™ (BD Bioscience, San Jose, CA, USA) was added during the last 5 h of incubation. Cells were surface stained with either anti-CD4 or anti-CD8 antibody (BD Bioscience), followed by intracellular staining with anti-IFN-γ antibody (BD Bioscience). Samples were analyzed using an LSRII Flow cytometer (BD Biosciences), and the cytometric data were analyzed using FlowJo software version 9.3.3 (Tree Star, Inc., Ashland, OR, USA).

### Hemagglutination inhibition assay

Sera collected from control and immunized mice were treated with receptor-destroying enzyme (RDE, Denka Seiken, Japan) at 37 °C overnight and tested against RG A/IN/05 virus by the standard hemagglutination inhibition (HI) assay with 1% horse red blood cells (RBCs). Briefly, 25 µl of 1× PBS was added to wells of a 96-well V-bottom plate (Corning, NY, USA). This was followed by adding 50 µl of RDE-treated sera to each column, which was then serially diluted. Of 4 HA units of RG A/IN/05 virus, 25 µl was added to each well and incubated at RT for 60 min. Finally, 50 µl of 1% standardized horse RBCs in PBS was added to each well and incubated at RT for exactly 60 min. The reciprocal serial dilutions of the sera that showed complete inhibition of hemagglutination were recorded as the HI titer.

### Statistical analysis

Statistical analyses were performed using GraphPad Prism 5.0 software (GraphPad Software, La Jolla, CA, USA). Groups were compared by one-way analysis of variance (ANOVA) followed by Bonferroni multiple comparison test. The Mann–Whitney *U*-test was used to determine significance of antibody (HI) titers. The log-rank (Mantel–Cox) test was used to compare percent survival among groups of mice. Data were presented as mean ± standard error of mean (SEM). All differences were considered statistically significant when the *p* value was ≤.05.

## Results

### GenJet™ and in vivo-jetPEI^®^ enhanced the H5N1 vaccine-induced antibody responses and memory B-cell responses

**Figure 1.**
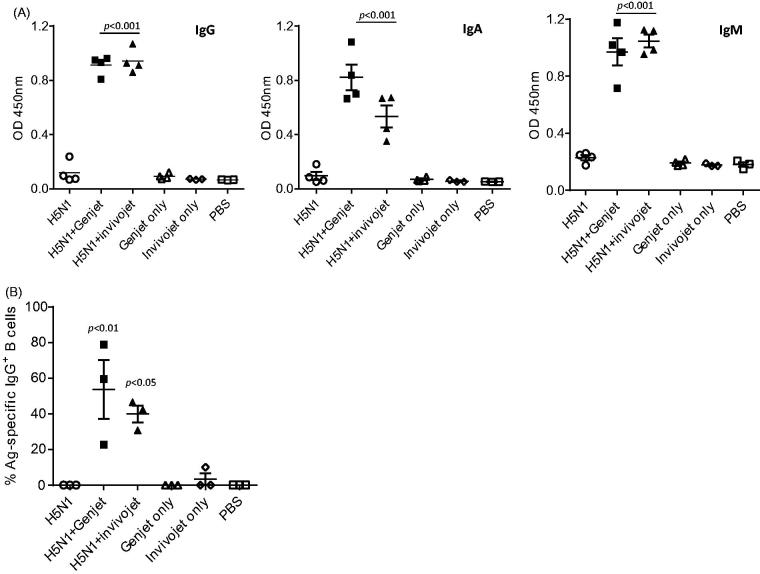
GenJet™ and *in vivo*-jetPEI enhances the H5N1 vaccine-induced systemic antibody responses and memory B-cell responses. Balb/c mice (5 mice/group) were intranasally administered with 3 µg of A/IN/05 vaccine with or without GenJet™ (10 µl/each mouse as described in previous study (Kulkarni et al., 2014)) or *in vivo*-jetPEI^®^ (0.6 μl/mouse according to manufacturer’s protocol). One month later, mice were boosted with the same vaccine formulations. The control mouse group received GenJet™, *in vivo*-jetPEI^®^ or PBS at both time points. (A) Three weeks after booster immunization, sera were collected and IgG, IgA and IgM antibodies against A/IN/05 were assessed by ELISA. (B) One week after booster immunization, the spleen were harvested and the frequency of A/IN/05-specific IgG^+^ ASCs in the spleen were measured by ELISPOT assay. The number of A/IN/05-specific IgG^+^ ASCs were normalized against the number of total IgG^+^ secreting ASCs and presented as % Ag-specific IgG^+^ B cells. The data are representative of two independent experiments (3–5 mice each group) and error bars represent SEM. One-way ANOVA with Bonferroni post-analysis was used to analyze differences among different groups. *p* < .05, *p* < .01 and *p* < .001 as compared to the H5N1 vaccine alone group.

Vaccines for protection against influenza virus infections should induce an optimal systemic antibody response. To quantitate the antibody responses, mice were immunized intranasally with a H5N1 monovalent vaccine including 3 µg of HA with or without cationic polymers (GenJet™ or *in vivo*-jetPEI^®^). Sera were collected at three weeks post-booster immunization to measure the antigen-specific IgG, IgM and IgA production by ELISA. Control mice were immunized with the formulations alone or PBS without antigen. As shown in Figure 1(A), sera from mice immunized with formulations only or PBS have no detectable levels of antibody responses. The H5N1 vaccine group also did not induce significant antibody responses, though one mouse had slightly elevated IgG and IgA production. However, when formulated with GenJet™ or *in vivo*-jetPEI^®^, H5N1 vaccine induced significantly higher IgG, IgA and IgM production in the sera as compared to the controls. We next examined the memory B-cell response in the spleen upon booster immunization by measuring the frequency of H5N1-specific ASCs by an ELISPOT assay. The frequency of H5N1-specific IgG ASCs in the spleens of mice immunized with formulations alone or PBS are low or undetectable (Figure 1(B)). Immunization with H5N1 vaccine alone did not increase the frequency of ASCs, while the addition of either GenJet™ (*p* < .01) or *in vivo*-jetPEI^®^ (*p* < .05) to the vaccine significantly increased the frequency of IgG-secreting cells in the spleen. In summary, these data suggest that intranasal administration of H5N1 vaccine formulated with GenJet™ or *in vivo*-jetPEI^®^ enhanced the serum antibody responses and memory B-cell responses in the spleen.

### GenJet™ and in vivo-jetPEI^®^ enhanced the H5N1-specific T-cell responses

**Figure 2.**
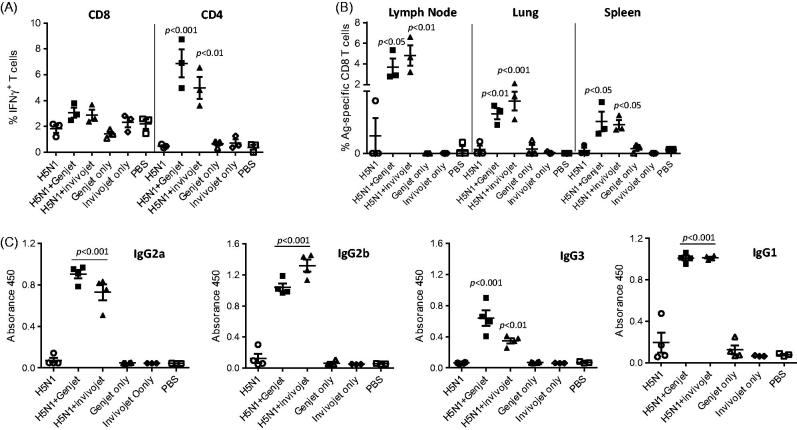
GenJet™ and *in vivo*-jetPEI^®^ enhanced the H5N1-specific T-cell responses. Balb/c mice (3–5 mice/group) were intranasally administered with A/IN/05 vaccine with or without GenJet™ or *in vivo*-jetPEI^®^ using prime-boost regimen as described in Figure 1. (A) One week after booster immunization, the lung tissues were harvested and single cell suspensions were prepared. About 10^6^ cells from the lung were stimulated *in vitro* with HA peptide for 6 h to examine the antigen-specific CD8 T-cell response; or with RG A/IN/05 virus at an MOI of 1 for 16 h to examine the antigen-specific CD4 T-cell response. GolgiPlug™ was added during the last 5 h of incubation. Cells were surface stained with anti-CD44, anti-CD4 or anti-CD8 antibody (BD Bioscience), followed by intracellular staining with anti-IFNγ antibody (BD Bioscience). The frequency of IFN-γ producing T cells in total activated T cells was presented. (B) One-week post-booster immunization, the draining lymph nodes, lungs and spleen tissues were harvested and the frequency of HA518-specific CD8 T cells in total activated CD8 T cells was stained using H-2K^d^/IYSTVASSL tetramer. (C) Three weeks after booster immunization, sera were collected and IgG2a, IgG2b IgG3 and IgG1 antibodies against A/IN/05 were assessed by ELISA. The data are representative of two independent experiments (3–5 mice each group) and error bars represent SEM. One-way ANOVA with Bonferroni post-analysis was used to analyze differences among treatments. *p* < .05, *p* < .01 and *p* < .001 as compared to the H5N1 vaccine alone group.

Optimal antibody responses against influenza virus infection require the help from T-cell immune responses. T-cell activation is initiated in the draining lymph nodes after resident DCs in lungs capture antigen and present processed antigens to the antigen-specific T cells in the lymph nodes. Activated T cells then migrate to sites of infection to execute their effector functions. IFN-γ is one of the most important cytokines produced by effector T cells. The activation of vaccine-specific CD4 and CD8 T cells in the lung was examined in mice immunized with H5N1 vaccine with or without formulations using prime-boost regimen. Lung tissues were harvested 1 week post-booster immunization and IFN-γ production by activated T cells in the lung was measured by intracellular cytokine staining. H5N1 vaccine alone did not increase the IFN-γ production in activated CD4 and CD8 T-cell subsets. H5N1 vaccine with formulations slightly increased the IFN-γ production among CD8 T cells, but the change did not reach statistical significance (Figure 2(A) and Supplementary Figure S1). However, co-administration vaccine with GenJet™ or *in vivo*-jetPEI^®^ significantly increased IFN-γ production by activated CD4 T cells (Figure 2(A) and Supplementary Figure S1).

Virus-specific cytotoxic CD8 T cells play a central role in the immune responses by eliminating virus infected cells. In response to H5N1 vaccination, antigen-specific CD8 T cells from the draining lymph nodes, spleens, and sites of infection (lungs) can be identified with the H-2K^d^/IYSTVASSL (HA518) tetramer. As shown in Figure 2(B), in mice immunized with H5N1 vaccine alone, the frequencies of HA518-specific CD8 T cells were very low in the lymph node and almost undetectable in the lung and spleen tissues. In contrast, GenJet™ or *in vivo*-jetPEI^®^-formulated-H5N1 vaccine remarkably increased the frequencies of HA518-specific CD8 T cells ∼1.5–10-fold in all tissues examined.

Virus-activated CD4 T cells can mature into distinct T_H_ cell types such as T_H_1, T_H_2 and T_H_17 (Hirahara and Nakayama, 2016). T_H_1 or T_H_2 subsets drive differential B-cell responses to secrete distinct immunoglobulin isotypes (Mosmann et al., 1986; Abbas et al., 1996; Mosmann and Sad, 1996). The isotypes of serum H5N1-binding antibodies from mice immunized with H5N1 vaccine with or without formulations were measured by ELISA. H5N1 vaccine alone elicited more IgG1 production than IgG2a, IgG2b and IgG3 as shown in Figure 2(C). However, GenJet™ and in vivo-jetPEI^®^ adjuvantation induced a mixed IgG1, IgG2a, IgG2b and IgG3 responses (Figure 2(C)). Therefore, these formulations significantly enhanced both the antigen-specific CD8 responses as well as CD4 T cell activation with mixed T_H_1 and T_H_2 responses.

### *GenJet™ and* in vivo*-jetPEI^®^ enhanced the protective immunity*

**Figure 3.**
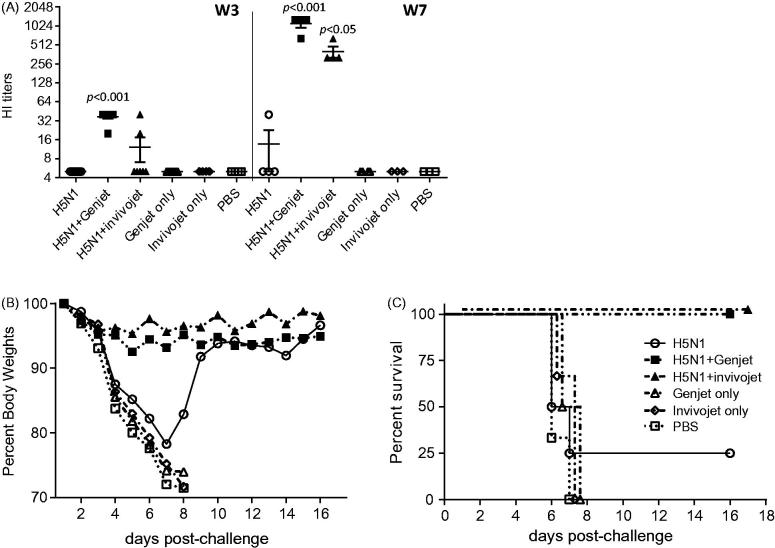
GenJet™ and *in vivo*-jetPEI enhanced the protective immunity. Balb/c mice (5 mice/group) were intranasally administered with A/IN/05 vaccine with or without GenJet™ or *in vivo*-jetPEI^®^ using the prime-boost regimen as described in Figure 1. (A) Sera were collected at the third week following primary immunization (W3) and booster immunization (W7). HI titers were measured against RG A/IN/05 virus. (b,c) Four weeks following booster immunization, mice were challenged with 5 × LD_50_ of RG A/IN/05 virus. Mice were weighed every day to monitor body weight changes (B) and mortality (C). Mice that lost more than 25% body weight were euthanized and scored as a fatality. One-way ANOVA with Bonferroni post-analysis was used to compare the percentage of body weight changes and the log-rank (Mantel–Cox) test was used to compare percent survival among groups of mice. *n* = 5 mice for each group from two independent experiments and the error bars represent SEM. *p* < .05, *p* < .01 and *p* < .001 as compared to the H5N1 vaccine alone group.

To investigate whether nasal administration of GenJet™ or *in vivo*-jetPEI^®^ could enhance the protective efficacy of an H5N1 vaccine, the production of sera virus-neutralizing antibody was measured by HI assay. Mice were immunized with a prime-boost regimen 4 weeks apart. Sera samples were collected at 3 weeks following primary immunization (week 3 sera) and booster immunization (week 7 sera). HI titers against RG A/IN/05 viruses were measured. Mice immunized with H5N1 vaccine alone did not induce detectable HI titers after primary vaccination (Figure 3(A)). However, mice immunized with GenJet™ or *in vivo*-jetPEI^®^-adjuvanted H5N1 vaccine developed low levels of HI titers even after a single immunization. Six out of seven mice immunized with GenJet™-formulated H5N1 vaccine showed an HI titer of 40, indicative of a protective response (Figure 3(A)). Three weeks post-booster immunization, only one mouse immunized with H5N1 vaccine alone had an HI titer of 40. However, all mice immunized with vaccine and formulations developed significantly higher HI titers (Figure 3(A)). These results suggest that GenJet™ and *in vivo*-jetPEI^®^ significantly enhanced the immunogenicity of H5N1 vaccine.

To further examine the protective efficacy of H5N1 vaccine, mice immunized with H5N1 vaccine formulated with or without GenJet™ or *in vivo*-jetPEI^®^ were challenged with a homologous RG A/IN/05 virus 4 weeks post-boost. All mice immunized with formulations alone or PBS showed significant body weight loss (Figure 3(B)) and succumbed to infection between Day 7 and 8 post-challenge (Figure 3(C)). Only one mouse out of four that received H5N1 vaccine alone survived the virus challenge, but showed significant weight loss (Figure 3(B,C)). In contrast, all mice immunized with the vaccine formulated with GenJet™ or *in vivo*-jetPEI^®^ survived challenge and no significant body weight loss was observed (Figure 3(B,C)). Therefore, both GenJet™ or *in vivo*-jetPEI^®^ significantly enhanced the protective efficacy of H5N1 vaccine.

## Discussion

The spread of highly pathogenic AIV in birds and domestic poultry, the emergence of novel virus strains, and the increasing number of their direct transmission to humans have raised significant concern about the avian influenza pandemic threat (Poovorawan et al., 2013). Vaccination to prevent infection is no doubt the most cost-effective public health strategy. Unfortunately, the current avian influenza vaccines are poorly immunogenic even with high dose or alternative immunization route as shown in animal studies and human clinical trials (FDA, 2007; Layton et al., 2011a, 2011b; Cao et al., 2016; Jones et al., 2017). The manufacturing capacity is also limited to produce enough vaccine in a pandemic situation. Adjuvants have been shown to provide great advantages in enhancing the immunogenicity of vaccine, such as dose sparing, increased vaccine efficacy in elder and immunocompromised populations, and a broader protective immunity (Reed et al., 2013). Aluminum salts are the most commonly used adjuvants in human vaccines (Reed et al., 2013). In recent years, influenza vaccines formulated with two squalene-based oil-in-water emulsions have been approved for use in human in the US and Europe for seasonal and pandemic influenza (FDA, 2015). Although both adjuvants hold great promise for enhancing the immunogenicity of vaccine, AS03 has been associated with narcolepsy (Ahmed et al., 2014) and these are proprietary adjuvants. Therefore, challenges remain for developing new adjuvants and alternative delivery systems for avian influenza vaccine.

Cationic polymers have gained tremendous attention in recent years as non-viral vectors in gene and protein delivery (Guy et al., 2001; Sanchez et al., 2001). PEI is the most widely used cationic polymer due to its strong DNA compaction capacity with an intrinsic endosomolytic activity (Di Gioia and Conese, 2009). The adjuvanticity of PEI has been demonstrated initially with viral subunit soluble glycoprotein antigens by Wegmann et al. (2012). The potency of gene delivery to the lung tissue by PEI (Di Gioia and Conese, 2009) and its adjuvanticity for H9N2 influenza whole inactivated virus in mouse model have been shown in earlier studies as well (Qin et al., 2015). Consistent with these findings, our data indicated that two efficient transfection reagents, GenJet™ and *in vivo*-jetPEI^®^, significantly increased serum antibody (IgG, IgA and IgM) responses when administerred together with H5N1 vaccine. The development of B cell memory is the key for rapid antibody production in response to the same pathogen. Shortly after immunization, antibody-secreting plasmablasts are highly abundant in the spleen (Giesecke et al., 2014). Our study further demonstrated that, one week post-booster immunization, the mouse group that received vaccine formulated with GenJet™ or *in vivo*-jetPEI^®^ displayed significantly higher frequency of IgG ASCs in the spleen. However, no detectable antigen-specific IgG antibody producing cells were observed in the spleens of mice immunized with the H5N1 vaccine alone. Therefore, both GenJet™ and *in vivo*-jetPEI^®^ induced enhanced systemic antibody responses and the number of antibody-secreting B cells found in the spleen following intranasal immunization.

Effective antibody response is essential to prevent influenza virus infection by blocking virus entry, but the optimal antibody response and ultimate elimination of the virus-infected cells require effector CD4 and CD8 T cells. Qin et al. (2015) showed that the activation and proliferation of total CD4 and CD8 T cells were increased by vaccination with H9N2 vaccine formulated with PEI (Qin et al., 2015). Our study further characterized the antigen-specific T cell response in mice immunized with H5N1 vaccine with or without GenJet™ or *in vivo*-jetPEI^®^. Both formulations substantially increased the frequency of antigen-specific cytotoxic CD8 T cells in the draining lymph nodes where T-cell activation was initiated. Activated T cells migrate from LN to the lungs where they eliminate virus-infected cells. After clearance, immunological memory is established both locally and systemically. The ability of CD8 T cells to recognize conserved viral genes is critical in conferring heterosubtypic immunity (Sridhar, 2016). In addition to the increased frequency of antigen-specific CD8 T cells, IFN-γ production by CD8 T cells was also slightly increased in the spleens of mice immunized with the formulated vaccine. Similar to CD8 T cells, the frequency of IFN-γ producing CD4 T cells were increased in mice that received the H5N1 vaccine formulated with GenJet™ or *in vivo*-jetPEI^®^. CD4 T-cell induction is required for the generation of protective antibody responses by B cell and expansion of antiviral CTL responses (Leist et al., 1987; Matloubian et al., 1994). Serum IgG isotype profiles were further characterized and the results showed that vaccine alone elicited more IgG1 (T_H_2 type response) production than IgG2a, IgG2b and IgG3 (T_H_1 type response), whereas the H5N1 vaccine formulated with GenJet™ or *in vivo*-jetPEI^®^ induced a mixed T_H_1 and T_H_2 response. Wegmann et al. (2012) and Qin et al. (2015) both demonstrated that PEI-adjuvanted vaccines enhanced the innate responses including antigen uptake, DC migration, and maturation which subsequently increased the humoral and cellular immune responses.

Hemagglutinin (HA) is the major surface protein of the influenza A virus which mediates virus binding and entry into cells. Therefore, the induction of antibody responses to HA are crucial. In the presence of GenJet™ or *in vivo*-jetPEI^®^, the H5N1 vaccine induced significantly higher HI titers even after single immunization while the vaccine alone failed to induce any detectable levels of HI titers. Upon booster immunization, only one out of four mice that received vaccine alone showed a HI titer above 40. However, all mice that received the vaccine formulated with GenJet™ or *in vivo*-jetPEI^®^ displayed 100–300-fold higher HI titers and subsequently were completely protected from homologous virus challenge.

In summary, GenJet™ and *in vivo*-jetPEI^®^ formulated H5N1 vaccine elicited significantly higher serum antibody responses and antigen-specific T- and B-cell responses. The neutralizing antibody responses against HA were also significantly increased in mice immunized with the vaccine plus formulations as compared to the vaccine alone groups. These enhanced immune responses culminated in enhanced protection against challenge with a homologous H5N1 virus. Both GenJet™ and *in vivo*-jetPEI^®^ have potential applications as adjuvants for poorly immunogenic influenza vaccines for nasal delivery to prepare for a H5N1 pandemic.

## Supplementary Material

IDRD_Cao_et_al_Supplemental_Content.pdf
