# Supplementary figures and images for: Nasal delivery of H5N1 avian influenza vaccine formulated with GenJet™ or in vivo-jetPEI® induces enhanced serological, cellular and protective immune responses
Source: Drug Deliv. 2018 Mar 15;25(1):773–9. doi: 10.1080/10717544.2018.1450909 (PMC6058713; doi:10.1080/10717544.2018.1450909)

Supplementary Figure 1

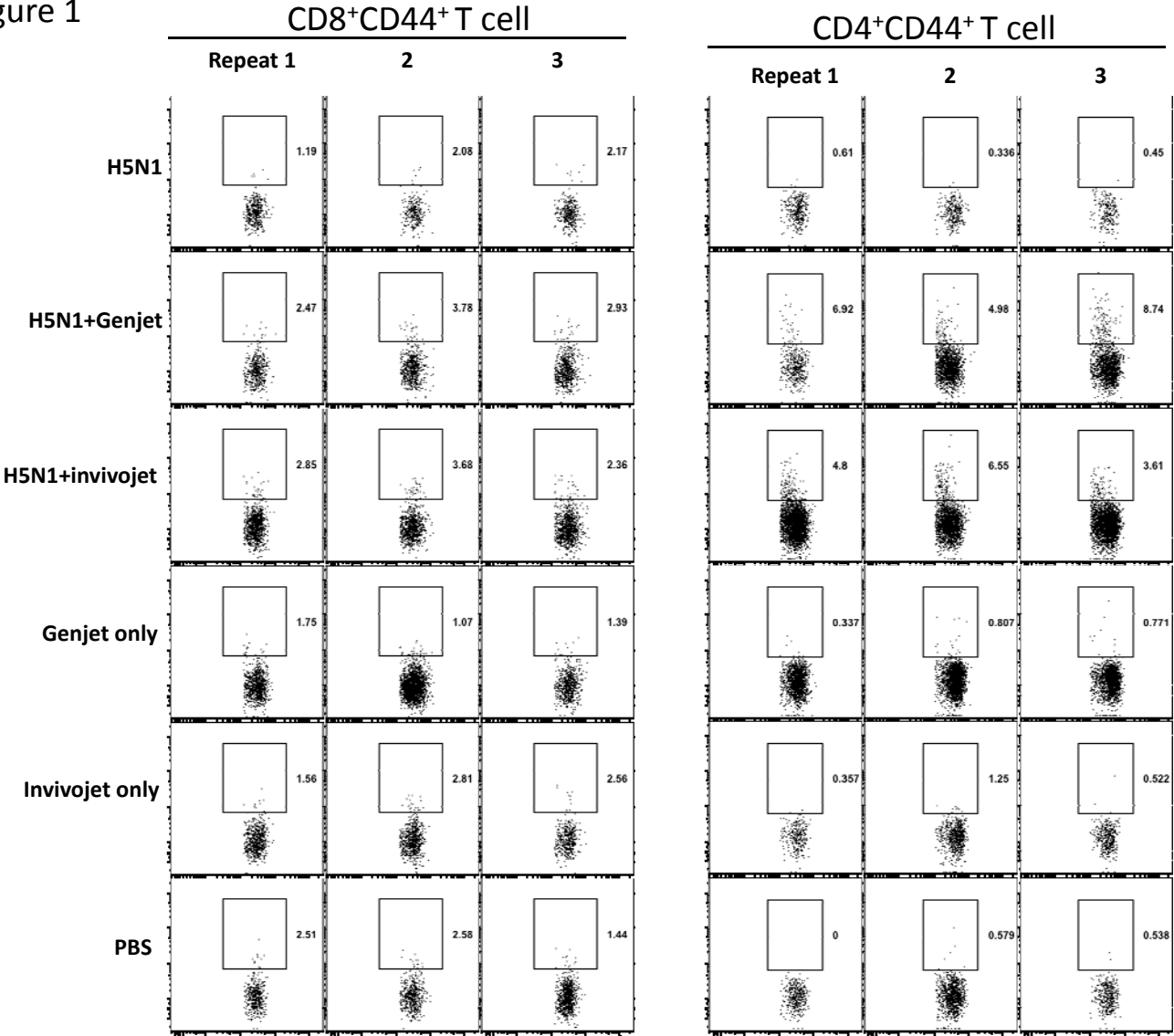

Supplement: IDRD_Cao_et_al_Supplemental_Content.pdf [file IDRD_A_1450909_SM1313.pdf]
